# *GmGSTU23* Encoding a Tau Class Glutathione S-Transferase Protein Enhances the Salt Tolerance of Soybean (*Glycine max* L.)

**DOI:** 10.3390/ijms24065547

**Published:** 2023-03-14

**Authors:** Xingang Li, Yuanting Pang, Yiwang Zhong, Zhandong Cai, Qibin Ma, Ke Wen, Hai Nian

**Affiliations:** 1 The State Key Laboratory for Conservation and Utilization of Subtropical Agro-Bioresources, South China Agricultural University, Guangzhou 510642, China; 2 Guangdong Laboratory for Lingnan Modern Agriculture, Guangzhou 510642, China; 3 The Guangdong Subcenter of the National Center for Soybean Improvement, College of Agriculture, South China Agricultural University, Guangzhou 510642, China; 4 Key Laboratory of Vegetable Biology of Hainan Province, Vegetable Research Institute of Hainan Academy of Agricultural Sciences, Haikou 570228, China; 5 Beijing Key Laboratory of Growth and Developmental Regulation for Protected Vegetable Crops, Department of Vegetable Science, College of Horticulture, China Agricultural University, Beijing 100193, China; 6 Hainan Yazhou Bay Seed Laboratory, Sanya Nanfan Research Institute, Hainan University, Sanya 572025, China

**Keywords:** soybean, glutathione transferase, salt stress, glutathione

## Abstract

Salt stress has a detrimental impact on crop yield, quality, and profitability. The tau-like glutathione transferases (GSTs) represent a significant group of enzymes that play a crucial role in plant stress responses, including salt stress. In this study, we identified a tau-like glutathione transferase family gene from soybean named *GmGSTU23*. Expression pattern analysis revealed that *GmGSTU23* was predominantly expressed in the roots and flowers and exhibited a concentration–time-specific pattern in response to salt stress. Transgenic lines were generated and subjected to phenotypic characterization under salt stress. The transgenic lines exhibited increased salt tolerance, root length, and fresh weight compared to the wild type. Antioxidant enzyme activity and malondialdehyde content were subsequently measured, and the data revealed no significant differences between the transgenic and wild-type plants in the absence of salt stress. However, under salt stress, the wild-type plants exhibited significantly lower activities of SOD, POD, and CAT than the three transgenic lines, whereas the activity of APX and the content of MDA showed the opposite trend. We identified changes in glutathione pools and associated enzyme activity to gain insights into the underlying mechanisms of the observed phenotypic differences. Notably, under salt stress, the transgenic Arabidopsis’s GST activity, GR activity, and GSH content were significantly higher than those of the wild type. In summary, our findings suggest that *GmGSTU23* mediates the scavenging of reactive oxygen species and glutathione by enhancing the activity of glutathione transferase, thereby conferring enhanced tolerance to salt stress in plants.

## 1. Introduction

Soil salinization is widely distributed in more than 60% of countries or regions, causing severe food and economic problems worldwide [1,2,3]. According to statistics, about 7% of the fundamental arable land in China is saline soil and has a trend of increasing yearly [4]. In saline soil, excessive sodium and chloride ions are released into the soil which are actively absorbed by plants, inhibiting seed germination and plant growth and development [5]. Salt stress can seriously affect crops’ yield, quality, and benefit, and it has become one of the principal abiotic stresses and limiting factors affecting crop production and agricultural development [5,6]. In order to resist the damage of salt stress, different salt tolerance mechanisms exist in crops [7,8,9]. Plant salt tolerance mechanisms mainly include ion homeostasis, osmotic adjustment, cell wall and membrane lipid modifications under salt stress, and changes in antioxidant-related enzymes [9,10,11].

Enzymes play an irreplaceable role in the normal life activities of plants. Currently, several families of salt stress tolerance genes have been identified in plant species [12,13,14,15]. Glutathione S-transferase (GST) is a ubiquitous, large, and diverse gene family [16,17,18]. *Arabidopsis*, rice, and soybean genomes contain 28, 41, and 59 family members (PTHR11260), respectively [18,19,20,21]. According to amino acid sequence similarity, these genes were classified into five subcategories of tau, phi, zeta, theta, and lambda [22,23,24]. Glutathione S-transferases (GSTs) catalyze the binding of glutathione to toxic biological heterologous substances or oxidation products to facilitate their metabolism, regional isolation, or elimination [25,26]. Their promoter contains MYB and MYC domains and at least one conservative protein domain (GST_NTER, GST_CTER). In its classical reaction mode, GSTs catalyze the transfer of glutathione (GSH) to a coenzyme (R-X) containing an active electrophilic center to establish a polar S-glutathione reaction product (R-SG) [22,25,26]. Recent studies have shown that GST protein is essential in multiple metabolic reactions [12,24,27]. It has been reported that GST protein in plant roots increases the biological nitrogen fixation efficiency of legume nodules [28,29,30]. Wen et al. found that GST protein might be involved in coupling exogenous glutathione with chromium ions to alleviate the toxicity of heavy metals [31]. In addition, GST protein may be involved in crop herbicide metabolism and selection, and has been used in the soybean and maize seed industries [24,32,33,34].

Tau class glutathione transferase is a vital enzyme in plant cells that plays a significant role in plant antioxidant defenses and metabolic regulation. It is of utmost importance for improving plant stress resistance, growth, and development. Recent research has demonstrated that regulating the expression and activity of tau glutathione transferases can significantly enhance plant adaptability to various stress environments, such as high salt, low temperature, and drought [35,36,37]. For example, the research by Cao et al. proved that *CsGSTU19* was involved in the defense of tea trees against temperature stress [36]. In *Medicago ruthenica* and *Medicago sativa*, *MruGSTU39* detoxified ROS under drought stress by up-regulating GST and glutathione peroxidase activities [37]. In addition, some GST genes, such as *PpGST*, *OsGSTL2*, and *PtGSTU51*, were also isolated and conferred heavy metal tolerance to *Pyrus pyrifolia*, *Oryza sativa*, and *Populus tomentosa*, respectively [38,39,40]. Moreover, the GST genes were also identified as necessary in the physiological and molecular mechanisms of plant resistance to salt stress. The salt tolerance of transgenic plants was enhanced after the GST gene of *Suaeda salsa* was heterologously transformed into *Arabidopsis thaliana* [41]. In a study of tomato *LeGSTU2* gene function, transgenic plants expressing tomato glutathione S-transferase showed enhanced resistance to salt and drought stress [42]. Similarly, *GsGST*, *PtGSTF1*, and *CsGSTU8* have been reported to have similar functions in *Glycine soja*, *Pyrus pyrifolia* and *Camellia sinensis* [43,44,45]. In particular, co-expression of the *Suaeda salsa* glutathione S-transferase and catalase in transgenic rice may have a synergistic effect in response to salt stress [46].

Soybean (*Glycine max*) is one of the most substantial oil and protein crops in the world and participates in the human diet and food industry as an essential raw material [47,48,49,50]. Salt stress will have a severe toxic effect on each growth stage of soybean plants, which will eventually leading to the loss of soybean yield and the reduction of soybean quality [51,52,53]. Predecessors studied the molecular mechanisms of soybean salt tolerance, and some genes were identified such as *GmPAL1.1*, *GmCHX1*, *GmSALT3*, *GmHKT1;4*, and *GmbZIP44* [54,55,56,57,58]. Meanwhile, the molecular mechanism of soybean GSTs under salt stress has not been thoroughly studied and identified. The primary objective of this investigation is to elucidate the involvement of glutathione S-transferase in the molecular mechanisms of plant salt tolerance. In the present study, a salt-responsive glutathione S-transferase gene, named *GmGSTU23*, was isolated from transcriptome-based analysis of changes in gene expression patterns after salt stress. The function of *GmGSTU23* was identified, and phenotypic analysis was performed on overexpressed transgenic lines from *Arabidopsis thaliana*. Our study postulated that *GmGSTU23* has a crucial role in plant responses to salt stress, ameliorating plant salt tolerance through its regulation of enzyme activity and the proportion of reduced glutathione in plants. The results of this study offer theoretical support in understanding the role of glutathione S-transferase in the molecular mechanisms of plant salt tolerance.

## 2. Results

### 2.1. Isolation and Bioinformatics Analysis of the GmGSTU23

**Figure 1:**
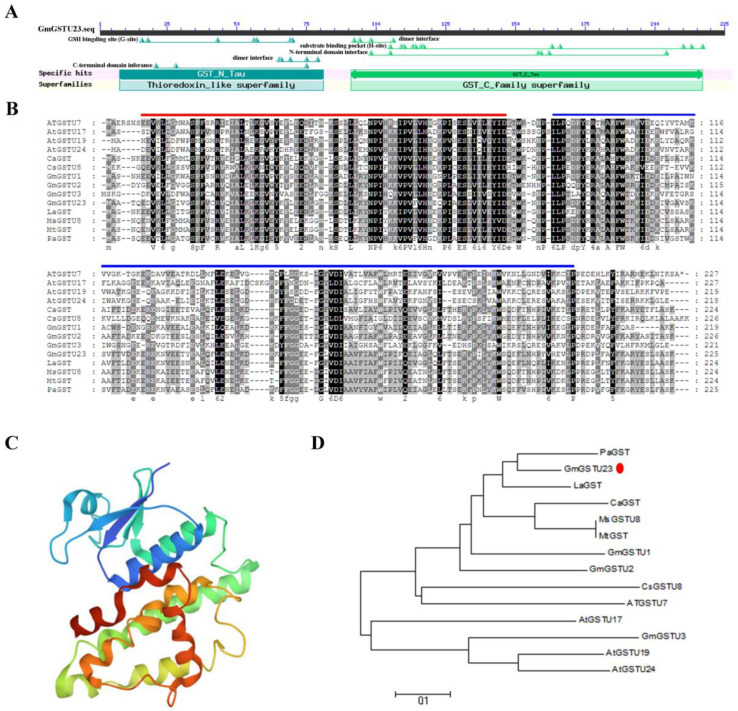
Bioinformatics analysis of *GmGSTU23*. (**A**) Conserved domain analysis of the GmGSTU23 protein sequence. (**B**) Sequence comparison of GmGSTU23 and its homologues. GmGSTU23 and its homologs both contain an N-terminal domain (red line) and a C-terminal domain (blue line). (**C**) Prediction of the GmGSTU23 protein structure. The protein structure modeling template is [PDB c6ghfA]. One color represents one protein fold. (**D**) Phylogenetic tree of GmGSTU23 and GST members in other species.

Based on the identification of salt-tolerant soybean material resources (unpublished data), a salt-induced gene encoding glutathione S-transferase was isolated from the high salt-tolerant soybean variety Guizao 1 (Table S1). A BLAST search in the soybean reference genome Wm82.a2.v1 identified the gene as *Glyma.07G139700* which was named *GmGSTU23* and belongs to the GST tau subfamily based on previous studies [59]. The soybean genome database showed that the full-length genomic sequence of *GmGSTU23* consisted of two exons and one intron, with a DNA length of 1066 bp, including a 678 bp CDS sequence, which encodes a peptide 226 amino acids in length with an isoelectric point of 5.39 (Table S1, Figure 1A). The amino acid sequence of GmGSTU23 was aligned with the GST members of other plant species. GmGSTU23 shares a degree of homology with members of the GST family from *Phaseolus vulgaris*, *Phaseolus vulgaris*, *Medicago sativa*, *Cicer arietinum*, and *Arabidopsis thaliana*, with the highest identity being with PvGST (Figure 1B). The Pfam database shows that GmGSTU23 has two typical conservative domains called GST-N and GST-C, representing the typical domain characteristics of GST family (Figure 1A,B). The predictions of the GmGSTU23 protein structural model showed that it has seven protein folds (Figure 1C). Subcellular localization predictions suggest that expression of GmGSTU23 may be located in the cytoplasm. Phylogenetic analysis revealed that GmGSTU23 homologs of legume species were clustered, and there were several homologous genes in the dicotyledonous plant *A. thaliana* (Figure 1D).

### 2.2. Expression Pattern Analysis of the GmGSTU23

**Figure 2:**
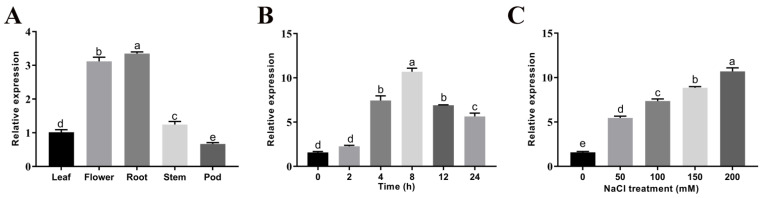
Determination of *GmGSTU23* expression patterns. (**A**) The relative expression of *GmGSTU*23 in different organs of soybean plants. (**B**) Time-dependent expression pattern of *GmGSTU23.* The plant materials were collected after 0, 2, 4, 8, 12, and 24 h in 200 mM NaCl. (**C**) Dose-dependent expression pattern of *GmGSTU23*. The seedings were measured after exposure to 0, 50, 100, 150, and 200 mM NaCl for 8 h. Different letters above the bars indicate significant differences among the treatments (*p* < 0.05).

In different plant organs, the expression of *GmGSTU23* was mainly concentrated in the flower and roots, while the expression in the leaf, stem, and pod was low (Figure 2A). In the experiment of detecting the response to time, *GmGSTU23* showed a significant upward trend after 4 h of NaCl treatment, and the expression level increased significantly with the treatment time and reached the maximum expression at 8 h, and then began to decline (Figure 2B). In addition, as shown in Figure 2C, the expression of *GmGSTU23* in soybean tissues significantly increased with the increase in NaCl concentration after exposure to salt for 8 h.

### 2.3. Generation and Detection of the GmGSTU23 Overexpression Lines

**Figure 3:**
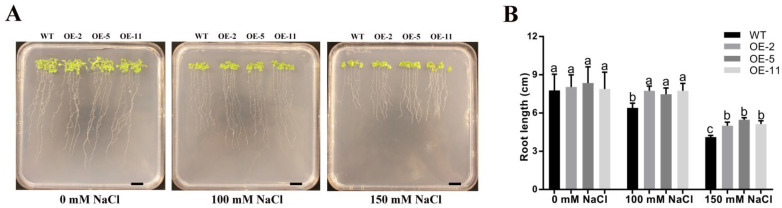
Overexpression of *GmGSTU23* relieved the inhibition of root elongation by salt stress. (**A**) Phenotypes of the *GmGSTU23* transgenic lines in plate culture. (**B**) Analysis of root elongation of seedlings. Scale bar = 1 cm. The bars represent the average ± SD of three replicates. Different letters above the bars indicate significant differences among the treatments (*p* < 0.05). WT, wild type; OE-2, OE-5, OE-11: *GmGSTU23* transgenic lines.

**Figure 4:**
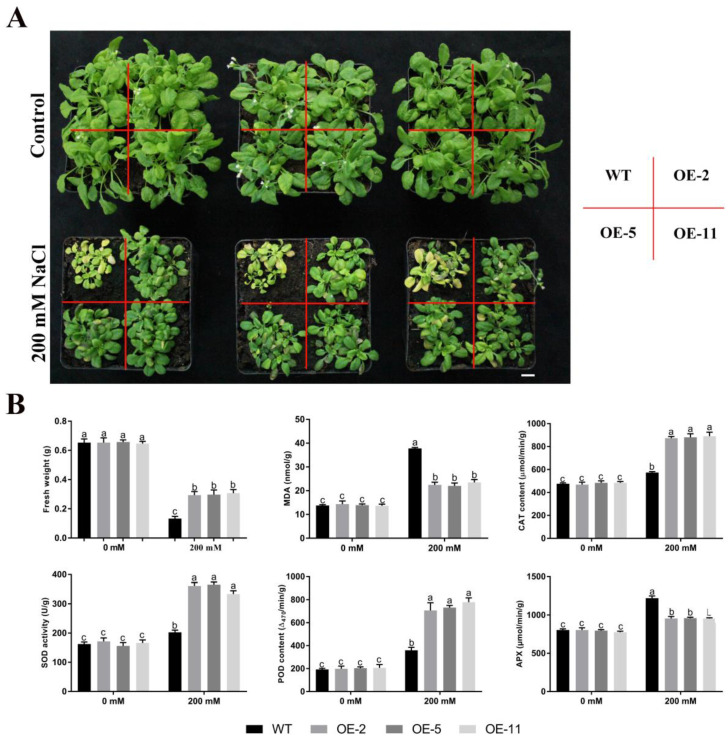
Overexpression of *GmGSTU23* in transgenic plants enhances phenotypic and antioxidant enzyme activity under salt stress. (**A**) Phenotypes of the *GmGSTU23* transgenic lines in soil culture. (**B**) Analysis of fresh weight, MDA, CAT, SOD, POD, and APX in the WT and transgenic lines. Scale bar = 1 cm. Different letters above the bars indicate significant differences among the treatments at *p* < 0.05.

To explore the function of *GmGSTU23* in plant salt stress responses, the coding sequence of *GmGSTU23* was inserted into the expression vector *pTF101* containing a strong CaMV 35S promoter, and the transgenic plant was obtained by the floral dip method. A total of 11 overexpressed lines were selected by herbicide identification and the expression levels of *GmGSTU23* in the different lines were determined by qRT-PCR (Figure S1). Then, three T_3_ generation transgenic lines (OE-2, OE-5, and OE-11) with high expression levels were selected for studying the salt tolerance phenotype and physiological analysis (Figure S1, Figure 3 and Figure 4).

### 2.4. Overexpression of GmGSTU23 Confers Phenotypic Tolerance to Salt Stress

To evaluate the salt tolerance of the *GmGSTU23* transgenic lines, the salt dose response phenotype of the seedlings was analyzed in agarose plate and soil culture. As shown in Figure 3A, the root elongation of wild-type and transgenic *A. thaliana* was inhibited to varying degrees under 100 mM or 150 mM NaCl treatment in the plate culture experiment. However, the inhibition of salt stress on the overexpression lines was significantly lower than that in the wild type (Figure 3B). Specifically, under the treatment of 150 mM NaCl, the root lengths of WT and *GmGSTU23* transgenic seedlings were 4.1 cm and 5.2 cm, respectively (Figure 3B). In the long-term salt stress test in soil culture, 15-day-old plants were continuously irrigated with 200 mM NaCl solution for 20 days. The phenomena of yellowing of plant leaves and stunted growth were revealed under salt stress. It was observed that the growth of wild-type plants was severely affected by salinity, while the growth of the transgenic plants was less affected (Figure 4A). Compared with the condition without salt stress, the fresh weight of WT and *GmGSTU23* transgenic lines under 200 mM NaCl treatment was decreased by 81.2% and 53.1%, respectively (Figure 4B). In addition, the content of malondialdehyde in the wild-type strain was significantly higher than that in transgenic lines under salt stress, which reflected the degree of membrane peroxidation (Figure 4B). The data on antioxidant enzyme activities showed that there was no significant difference between transgenic and wild-type plants in the absence of salt stress. Under salt stress, the activities of SOD, POD, and CAT in the wild type were significantly lower than those of the three transgenic lines, while the activity of APX showed the opposite situation (Figure 4B).

### 2.5. Changes of Glutathione Pool Composition and Related Enzymes under Salt Stress

**Figure 5:**
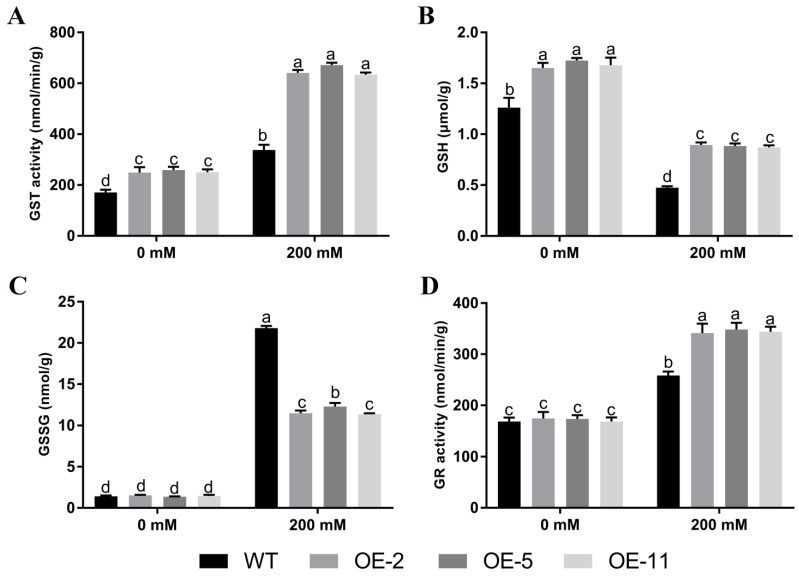
Glutathione pool composition and related enzymes. (**A**–**D**) Changes in GST (**A**), GSH (**B**), GSSG (**C**), and GR (**D**) contents of 15-day-old wild-type and transgenic *Arabidopsis thaliana* after 20 days of treatment with 0 or 200 mM NaCl solution. Different letters above the bars indicate significant differences among the treatments (*p* < 0.05).

To analyze the underlying causes of the phenotypic differences, glutathione pools and the associated enzyme activity changes were determined. Under salt stress, GST activity, GR activity, and GSSG content were increased, while GSH content showed the opposite effect (Figure 5). The analysis between wild-type and transgenic plants showed that the GST activity, GR activity, and GSH content of transgenic Arabidopsis thaliana were significantly higher than those of the wild-type strain under 200 mM NaCl treatment (Figure 5A,C,D). Specifically, there were significant differences in GST activity and GSH content in the absence of salt stress (Figure 5A,B).

## 3. Discussion

Salt stress is one of the abiotic stresses that has the most significant impact on agriculture. It can severely reduce the yield and quality of crops by destroying the homeostasis of plant ions, osmosis, and reactive oxygen species [5,6,7,10,11]. During their long evolution, plants have developed many responses to salt stress, such as calcium signals and glutathione-anti-circulating pathways to maintain cell homeostasis and eliminate reactive oxygen species [7,60]. The mechanisms of salt tolerance-related genes have been identified in some species, such as *AtHKT1;1, AtHAK5, OsLAX, OsABCB, SOS2, SnRK2, GmDof41, SlWRKY23,* etc., and these genes have endowed plants with different degrees of tolerance to salt stress [61,62,63,64,65,66,67]. The ascorbate–glutathione (ASA-GSH) cycle is widespread in living organisms and plays an essential role in plant oxidative defenses and abiotic stress responses [31,68]. During this cycle, GSTs, a family of proteins widely distributed in various organisms, plays a crucial role in detoxification [69]. Specifically, its primary function is to catalyze the coupling of some electrophilic groups with the thiols of reduced glutathione, to change its hydrophobicity so that it can easily cross the cell membrane to the extracellular space after being decomposed, thus achieving detoxification [70,71]. According to previous genome-wide identification reports of the GST family, it has been found that it has functions related to oxidative stress responses to abiotic stress [17,72,73,74,75]. *AtGSTF8*, *AtGSTU19*, *and AtGST1* relieved salt stress by maintaining redox homeostasis in *A. thaliana* roots [76,77]. The glutathione S-transferase gene has been isolated in a study in wild-type soybeans, and transgenic plants showed a higher tolerance to salt at the seedling stage than wild-type plants [43]. Meanwhile, the molecular mechanisms of soybean GST family genes under salt stress have been rarely reported and are unclear.

In this study, a salt-responsive glutathione S-transferase gene, defined as *GmGSTU23*, was identified as a differentially expressed gene under salt stress. Through sequence alignment and conservative domain analysis, we found that *GmGSTU23* shares a high degree of homology with the identified tau subfamily genes, particularly with *PaGST* (Figure 1A,D). These results indicated that the GST family genes are highly conserved among different species and may have similar functions in response to environmental stress [17,73]. Analysis of the promoter of *GmGSTU23* revealed a total of 3 ABREs (ABA-responsive elements), 2 LTREs (low-temperature responsive elements), 17 MYB-responsive elements, and 20 MYC-responsive element cis-counterparts. Among them, ABRE is mainly involved in the regulation of ABA response or drought expression [78,79]. Based on these results, it was speculated that *GmGSTU23* might be associated with abiotic stress responses in plants. Due to the existence of LTREs, some studies have suggested that *GmGSTU23* also encodes a heat shock protein in soybeans, which may respond to high-temperature stress, and further studies are needed [80].

The orthologous gene of *GmGSTU23* in *A. thaliana* is *At3g09270* with 80% similarity, which is thought to be involved in the early responses of *A. thaliana* cells to cadmium exposure [19,81]. Although it is similar to the GST family genes of other species in structure such as in the conservative domain, *GmGSTU23* may have a unique expression pattern and molecular function (Figure 1 and Figure 2). In some previous studies, the expression patterns of *GmGSTU23* were localized in the roots, especially in the root hair [82]. Meanwhile, our results showed that the expression of *GmGSTU23* is not confined to the roots, but is expressed in other organs of the plant, especially at high levels in the flowers (Figure 2A), which is similar to the results of some previous reports [83]. After 8h of salt treatment, the expression of *GmGSTU23* reached a peak, which suggested that *GmGSTU23* might be regulated by some transcription factors and may indirectly participate in plant salt stress responses (Figure 2B). The 3D structure model of *GmGSTU23* did not have a transmembrane domain, and its subcellular localization was predicted to be cytoplasmic. Therefore, it is speculated that GmGSTU23 was expressed in the cytoplasm to catalyze the binding of GSH and ROS (Figure 1 and Figure 2).

In order to further identify the mechanism of *GmGSTU23* in enhancing the tolerance of plants to salt stress, we obtained transgenic *A. thaliana* lines and conducted a salt stress treatment experiment. Compared with the control, the growth of wild-type and transgenic *A. thaliana* was inhibited under salt stress, and the leaves became withered and yellow (Figure 3A and Figure 4A). The results showed that under salt stress, the root length and fresh weight of transgenic *A. thaliana* were significantly higher than those of wild type (Figure 3B and Figure 4B). Malondialdehyde is generally considered to be an indicator of the degree of cell membrane peroxidation and the strength of plant responses to adverse conditions [84]. Malondialdehyde content in the transgenic lines was lower than in wild type, indicating that they were less damaged by ROS (Figure 4B) and that the overexpression of *GmGSTU23* enhanced the tolerance of plants to salt stress. The content of antioxidant enzymes in the transgenic *A. thaliana* and wild-type lines was also determined to study its response mechanism to salt stress (Figure 4B), and the results were similar to those of previous studies [39,40,41]. Some antioxidant enzymes such as SOD, POD, CAT, and APX can eliminate ROS in cells and reduce the generation of hydrogen peroxide, thereby enhancing salt tolerance [85]. We speculated that the *GmGSTU23* transgenic *A. thaliana* might induce changes in antioxidant enzymes through some pathways to improve the plant’s tolerance to salt stress. The measurement of glutathione pool composition and related enzymes showed that the GST activity and GSH content of the transgenic lines were significantly higher than those of the WT strain, regardless of the presence of salt stress (Figure 5). Therefore, it is speculated that *GmGSTU23* directly enhances the activity of glutathione transferase and participates in the clearance of reactive oxygen species with glutathione, thereby enhancing the tolerance of plants to salt stress [31,33,84]. However, little is known about the interactions between *GmGSTU23* and other transcription factors, which will need further exploration. Therefore, we will use a yeast library, RNA-Seq, and other techniques to study the function of the *GmGSTU23* gene in future studies to increase our understanding of the salt tolerance mechanisms in plants.

## 4. Materials and Methods

### 4.1. Plant Materials and Growth Conditions

The sterilized seeds of soybean variety Guizao 1, obtained from the South China Agricultural University (Guangzhou, China), were selected and sowed in vermiculite. After germination, the seedlings with consistent growth were selected and transferred to a hydroponic system (pH 6.8) with modified Hoagland nutrient solution at 28/25 °C and a 14 h/10 h (light/dark) photoperiod, as described by Ke Wen et al. [86]. The nutrient solution was rejuvenated daily. In the soil culture experiment, the plant organs including the roots, stem, leaf, flower, and pod were collected at the soybean V6 stage and then quickly frozen in liquid nitrogen and stored in an ultra-low-temperature refrigerator [86,87,88]. The experimental plants were grown in the basin with the nutrient soil in an environmentally controlled incubation chamber.

### 4.2. Analysis of Gene Expression Pattern

The plants were processed with the following treatments in the experiment to analyze the gene expression patterns in soybean plants (*G. max*) [86]. In order to determine the dose-dependent expression patterns, the seedings were cultivated in a solution with 0, 50, 100, 150, and 200 mM NaCl for 8 h. The plant materials were collected after 0, 2, 4, 8, 12, and 24 h in the 200 mM NaCl treatment to determine the time-dependent expression patterns. To determine organ-specific expression, the plant organs stored in the −80 °C refrigerator described in Section 4.1 were used for qPCR identification [31].

The total RNA was extracted from the plant samples using the RNA-easy Isolation Reagent (Vazyme, Nanjing, China) according to the manufacturer’s instructions and then reverse transcribed into cDNA using PrimeScript RT kit (Takara, Shiga, Japan). The gene expression level was evaluated by qPCR using the 2^−ΔΔct^ method using real-time PCR on a CFX96™ Touch Real-Time PCR System (Bio-Rad, Hercules, CA, USA) with SYBR Premix ExTaq™ II Mix (TaKaRa, Shiga, Japan) [86]. GmACTIN6 (GeneBnak Accession: AAK285830.1) was used as an internal control for three technical replicates. All primers were designed using the NCBI Primer tool (http://www.ncbi.nlm.nih.gov/, accessed on 4 February 2022) and are shown in Table S2.

### 4.3. Cloning and Bioinformatics Analysis of GmGSTU23

In this study, the cDNA of the soybean variety Guizao 1, reverse-transcribed from its mixed tissue RNA sample, was used to clone the gene sequence. *GmGSTU23* was extracted with PrimeScript RT Reagent Kit with gDNA eraser (TaKaRa, Shiga, Japan) by PCR using the specific primer pair: 5′-CGCATTCATACGCAGCAATCA-3′ and 5′-AGCAATAACTCAACAAGACAAGT-3′. The PCR cloning was performed with primers and cDNA using the following program: 5 min at 94 °C; 35 cycles of 30 s at 94 °C, 1 min at 54 °C, and 2 min at 72 °C; and 5 min at 72 °C for the final extension.

The sequence with the correct molecular size detected by agarose gel electrophoresis was integrated into the pLB vector (Tiangen Biotech, Beijing, China) and sent to a sequencing company (Shenggong, Guangzhou, China) for sequencing. The multiple protein sequence alignment of *GmGSTU23* with other glutathione S-transferases (GST) was performed using ClustalW2 (https://www.ebi.ac.uk/Tools/msa/clustalw2/, accessed on 15 January 2023). The tree of gene homology and molecular evolutionary genetics analysis was constructed using MEGA-X software via the Neighbor-Joining (NJ) method. The protein 3D structure of *GmGSTU23* was predicted by the phyre2 online prediction website (http://www.sbg.bio.ic.ac.uk/phyre2/html/page.cgi?id=index, accessed on 17 January 2023). The subcellular localization prediction was performed by the Cell-PLoc 2.0 online tool (http://www.csbio.sjtu.edu.cn/bioinf/Cell-PLoc-2/, accessed on 17 January 2023).

### 4.4. Vector Construction and Transgenic Lines Generation

To obtain transgenic *Arabidopsis thaliana* lines, the exonic region of *GmGSTU23* was amplified using gene-specific primers with *XbaI* and *SacI* sites (Table S2). *GmGSTU23* was then linearly cloned into the *pTF101.1* binary vector with a modified CaMV 35S promoter, which contained a phosphinothricin acetyltransferase (bar) resistance gene (phosphinothricin N-acetyltransferase, PAT) as selection marker for the transgenic line. The constructed vector was transformed into Agrobacterium strain GV3101 by electroporation (Gene Pulser Xcell™ Electroporation Systems, Hercules,CA, USA), and the target gene was transferred into the *A. thaliana* ecotype Columbia (Col-0) by flower dipping [89]. The T_0_ generation of *A. thaliana* was identified by overexpression by herbicide (Liberty^®^, Bayer, Leverkusen, Germany) spraying and PCR molecular identification. Molecular identification of the T_3_ transgenic line was performed using the 2 × Taq Plus Master Mix (Vazyme, Nanjing, China) according to the manufacturer’s instructions. The specific primers used for PCR were designed based on the vector and the gene sequence (Table S2).

### 4.5. Phenotypes Analysis of GmGSTU23 Transgenic Lines

To analyze the phenotypes of *GmGSTU23-*overexpressing (OE) and wild-type (WT) *A. thaliana* under salt stress, three T_3_ transgenic lines with high expression levels were selected for 1/2 MS agar plates and soil culture experiments. Specifically, the seeds sterilized with 10% sodium hypochlorite for 10 min were sown on 1/2 MS agar plates and vernalized at 4 ℃ in the dark for four days [86]. Subsequently, the plates were erected and placed in a growth chamber at 22–24 °C, with 60% relative humidity and a 16 h/8 h (light/dark) photoperiod until the roots grew to 1 cm. The seedlings with consistent growth were transferred to 1/2 strength MS agar medium containing different concentrations of NaCl (0 mM, 100 mM, 150 mM) for ten days. The length from the base of the rosette leaf to the top of the straight root was measured with a ruler, and the image was photographed with a Canon EOS 750d camera. In addition, T_3_ transgenic seeds and wild-type seeds (Columbia-0) after vernalization were planted in sterile substrate soil (Jiffy, Oslo, Norway) with a 16 h/8 h (light/dark) photoperiod in a GXZ-300D illuminated growth incubator (Jiangnan, Ningbo, China). The two-week-old seedlings were treated with 200 mM NaCl solution irrigation for 20 days. Then, the fresh weight and height of the plants were measured, and the leaf of the plants were quickly frozen in liquid nitrogen and stored in a refrigerator at −80 °C for the determination of various physiological indicators [43].

### 4.6. Physiological Analysis of GmGSTU23 Transgenic Lines

To analyze the physiology of the transgenic lines, 0.2 g of the flash frozen sample was accurately weighed, transferred to a centrifuge tube containing 1 mL of the sodium phosphate buffer, ground into a homogenate by a hand-held grinder, and centrifuged at 12,000 rpm for 15 min at 4 °C. Then, the absorbance of supernatant was measured at different wavelengths using a spectrophotometer (Shanghai Precision Instruments Co., Ltd., Shanghai, China, Model v-5800). Specifically, the absorbance values of glutathione S-transferase (GST), glutathione (GSH), oxidized glutathione (GSSG), glutathione reductase (GR, EC 1.6.4.2), malondialdehyde (MDA), peroxidase (POD, EC 1.11.1.7), superoxide dismutase (SOD, EC 1.15.1.1), peroxidase (CAT, EC 1.11.1.6), and ascorbate peroxidase (APX, EC 1.11.1.11) were measured at 340 nm, 412 nm, 412 nm, 532 nm, 470 nm, 450 nm, 510 nm, and 290 nm, respectively. The above physiological indexes were determined according to a previous method and using the corresponding kit (Grace Biotechnology, Suzhou, China) [31].

### 4.7. Statistical Analysis

The experiment was designed with random blocks, and there were at least three experiments and biological repetitions. The data are shown as the mean ± SE (standard error) of the three replicates. For statistical analysis, SPSS Statistics Ver. 22 (IBM, New York, NY, USA) and GraphPad Prism 6 (Boston, MA, USA) were used. One-way ANOVA and Student’s t-test were used for comparisons, and *p* ≤ 0.05 was the threshold for a significant difference.

## 5. Conclusions

In this study, a soybean gene *GmGSTU23* encoding a tau class glutathione transferase was identified. *GmGSTU23* was up-regulated under salt stress. The overexpression of *GmGSTU23* in *Arabidopsis thaliana* lines enhanced their tolerance to salt stress. Based on the physiological data and previous studies, we speculated that *GmGSTU23* might improve the activity of glutathione transferase and the clearance of ROS by GSH, thereby alleviating salt toxicity in plants. This study provides some support for understanding the mechanisms of plant regulation of salt tolerance.

## Data Availability

The data sets supporting the results of this study are included in the article. The RNA-seq data have been deposited into the NCBI Short Read Archive (SRA, https://www.ncbi.nlm.nih.gov/sra/, accessed on 4 February 2022) under accession number PRJNA867574.

## Supplementary Materials

The following supporting information can be downloaded at: https://www.mdpi.com/article/10.3390/ijms24065547/s1.
