# Supplementary material for: GmGSTU23 Encoding a Tau Class Glutathione S-Transferase Protein Enhances the Salt Tolerance of Soybean (Glycine max L.)
Source: Int J Mol Sci. 2023 Mar 14;24(6):5547. doi: 10.3390/ijms24065547 (PMC10058988; doi:10.3390/ijms24065547)
Supplement: Supplementary file 1 [file ijms-24-05547-s001.zip › ijms-2245777-SI.pdf]

*Supplementary Materials*

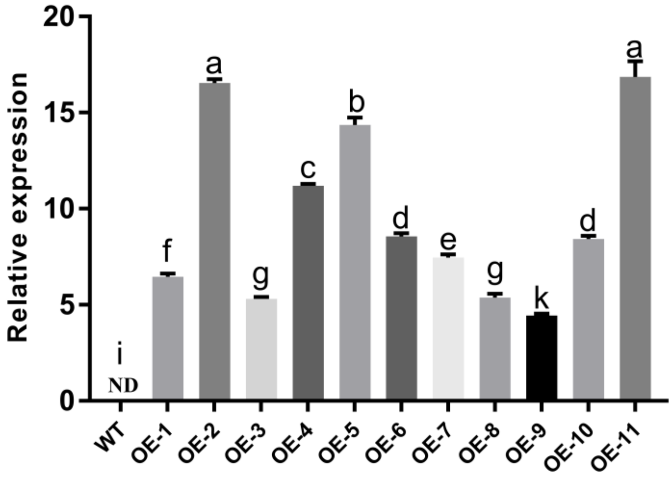


**Figure S1.** Generation and molecular confirmation of transgenic Arabidopsis thaliana. qRT-PCR identification of *GmGSTU23* transgenic Arabidopsis lines. WT: wild type; OE-1-OE-11: *GmGSTU23* transgenic Arabidopsis lines of T3 generation. ND: not detected. Data are means ± SD of three biological replicates. Different letters in the column indicate significant differences among the treat ments at $p < 0.05$.

**Table S1.** The sequencing information of *GmGSTU23*.

**CDS：**

ATGGCAGCTACTCAGGAAGATGTGAAGCTTTTGGGTATTGTGGGAAGCCCATTTGTGTG-
CAGGGTCCAGATTGCCCTTAAGTTGAAGGGAGTTGAATACAAATTTTTGGAAGAAAATTTGGG-
CAACAAGAGTGATTTGCTTCTCAAATACAACCCTGTTCACAAGAAGGTTCCAGTGTTT-
GTTCACAATGAGCAGCCCATAGCAGAGTCTCTTGTGATTGTTGAATACATTGATGAGA-
CATGGAAGAACAACCCCATCTTACCTTCTGATCCTTACCAAAGAGCCTT-
GGCTCGTTTCTGGTCCAAATTCATTGATGATAAGATTGTGGGTGCTG-
TATCGAAATCTGTTTTCACGGTTGATGAGAAAGAGCGTGAGAAGAATGTTGAAGAAACAT-
ATGAGGCTCTTCAGTTTCTTGAGAATGAGCTGAAGGACAAGAAGTTTTTTGGAGGAGAG-
GAATTTGGGTTGGTAGATATTGCTGCTGTCTTCATAGCATTTTGGATCCCAATTTTTCAG-
GAAATAGCAGGGTTGCAGTTATTCACCAGTGAGAAATTTCCTATACTCTACAAATGGAGCCAA-
GAATTCCTTAACCACCCTTTTGTGCACGAAGTCCTTCCTCCTAGAGACCCACTTTTTGCC-
TACTTCAAAGCCCGCTATGAAAGTCTTTCTGCTTCAAAATA

**cDNA：**

ATGGCAGCTACTCAGGAAGATGTGAAGCTTTTGGGTATTGTGGGAAGCCCATTTGTGTG-
CAGGGTCCAGATTGCCCTTAAGTTGAAGGGAGTTGAATACAAATTTTTGGAAGAAAATTTGGG-
CAACAAGAGTGATTTGCTTCTCAAATACAACCCTGTTCACAAGAAGGTTCCAGTGTTT-
GTTCACAATGAGCAGCCCATAGCAGAGTCTCTTGTGATTGTTGAATACATTGATGAGA-
CATGGAAGAACAACCCCATCTTACCTTCTGATCCTTACCAAAGAGCCTT-
GGCTCGTTTCTGGTCCAAATTCATTGATGA-
TAAGGTAACTCAACATTTCAAAAATCTTCTTAGTTTTCATGATTTGTGCTGATTTGTCAG-
CAAAACATCACGATGAAATCTATATATGTGAAATCTTTCTGGTGTG-
GAATATATATGTGAAATCTTTGAATATGTTAGAGAACTCAAAAGTCAACAGCCAACCATGAT-
TTTTTTTTAATGTATCAACTTTTTGTAAAACAATATTAGTGATTT-
GAAACTTTATGGAATCATATACTATGATTTTGGGA-
GAAATTTTATTTTATTTTACTATTTTTATCTGGGGTGGGGGGATTCTGTCACGTATTTGTTTC-
TATATAACTCGATCTAAATTCTGTTTGTCCTAATCACTTTATGAAATAATTACTAATAAA-
TATTGTGATTTGCGAAATCAGATTGTGGGTGCTGTATCGAAATCTGTTTTCACGGTTGATGA-
GAAAGAGCGTGAGAAGAATGTTGAAGAAACATATGAGGCTCTTCAGTTTCTTGA-
GAATGAGCTGAAGGACAAGAAGTTTTTTGGAGGAGAGGAATTTGGGTTGGTAGA-
TATTGCTGCTGTCTTCATAGCATTTTGGATCCCAATTTTTCAGGAAATAGCAGGGTTGCAG-
TTATTCACCAGTGAGAAATTTCCTATACTCTACAAATGGAGCCAAGAATTCCTTAAC-

| |
|---|
| CACCCTTTTGTGCACGAAGTCCTTCCTCCTAGAGACCCACTTTTTGCCTACTTCAAAGCCCGC-TATGAAAGTCTTTCTGCTTCAAAATAG |

**Table S2.** Primers used in the study.

| Primer Name | Primer Sequence (5′ to 3′) | Purpose |
|---|---|---|
| Clone-*GmGSTU23*-F | CGCATTCATACGCAGCAATCA | *GmGSTU23* Cloning |
| Clone-*GmGSTU23*-R | AGCAATAACTCAACAAGACACAAGT | |
| pTF101.1-*GmGSTU23*-F | gagaacacgggggactctagaATGGCAGCTACTCAGGAAGATGTG | *GmGSTU23*-pTF101.1 |
| pTF101.1-*GmGSTU23*-R | cgatcggggaaattcgagctcCTATTTTGAAGCAGAAAGACTTTCATAGC | |
| Detect-F | CCTTCGCAAGACCCTTCCTC | Molecular identification |
| Detect-R | TCATCGCAAGACCGGCAAC | |
| Actin-F | GCACCACCGGAGAGAAAATA | qRT-PCR |
| Actin-R | GTGCACAATTGATGGACCAG | |
| q*GmGSTU23*- F | GAGCAGCCCATAGCAGAGTC | |
| q*GmGSTU23*- R | AGATTTCGATACAGCACCCACA | |
